# Phage Therapy: Combating Evolution of Bacterial Resistance to Phages

**DOI:** 10.3390/v17081094

**Published:** 2025-08-08

**Authors:** Stephen T. Abedon

**Affiliations:** Department of Microbiology, The Ohio State University, Mansfield, OH 44906, USA; abedon.1@osu.edu

**Keywords:** antibiotics, bacteriophage therapy, community resistance, phage breeding, phage library, phage resistance, phage steering, polyphage, treatment resistance

## Abstract

Treatments for bacterial infections can be less effective due to toxicities, bacterial tolerance, or genetic resistance to antibacterial agents. The emphasis here is on combating genetic bacterial resistance to bacteriophages. Commonly described simply as phages, bacteriophages are the viruses of bacteria. As phage therapies, they are one of the oldest clinical treatments for bacterial infections. Thwarting bacterial evolution of resistance to phages, particularly during phage treatments, typically involves targeting more than one bacterial characteristic. This can be achieved serially, involving phage substitution after bacterial resistance has become problematic, something that is used especially during more personalized therapies. Substitution phages can be sourced in various ways. This includes as autophages, from phage banks, or via phage training—all as considered here—as well as through phage engineering. An alternative approach is preventing bacterial mutations from occurring at all. In addition, there is simultaneous targeting of multiple bacterial characteristics. These latter strategies include all of the following: using phages that target bacterial fitness or virulence determinants, employing individual phages that recognize multiple receptors, using phage cocktails, or applying phages in combination with antibiotics. This review discusses these different approaches for combating treatment resistance, highlighting various pros and cons.

## 1. Introduction

“Perhaps, the greatest challenge for successful implementation of phage therapy are concerns of the rapid acquisition of phage resistance and treatment failure.” Acton et al. [1].

Bacteriophages [2], often described simply as phages, are viruses that infect bacteria [3]. While most bacteria are harmless, bacteria that are pathogenic remain important health concerns. Infections caused by these bacteria—contrasting bacteria-caused intoxications—are typically treated using selectively toxic agents, most commonly antibiotics. Bacteriophages, however, are among the earliest discovered selectively toxic antibacterial agents.

The generally agreed-upon first-published treatments of patients using phages were in 1919 [4,5] and 1921 [6]. These preceded the first publication describing penicillin [7,8] by nearly a decade. It was another half a decade before penicillin was first tested clinically [9]. And well over two decades before penicillin, along with other antibiotics, became broadly clinically available [10,11,12]. This use of phages as antibacterial agents, especially in clinical practice, has been labeled ‘phage therapy’ or ‘bacteriophage therapy’ [13,14,15,16,17,18,19,20]. These are phrases, in this case as “Bacteriophage therapy”, dating back in the English-language literature to at least 1925 [21]. In addition, there are related antibacterial phage applications. Those include the removal of bacteria from foods or from inanimate surfaces (disinfection). Many of these latter strategies can be described as forms of biological control or biocontrol of bacteria [13,22,23,24,25,26,27,28] as well as bio-sanitization or bio-preservation [29], but which are considered here collectively as equivalent to ‘phage therapy’.

**Figure 1:**
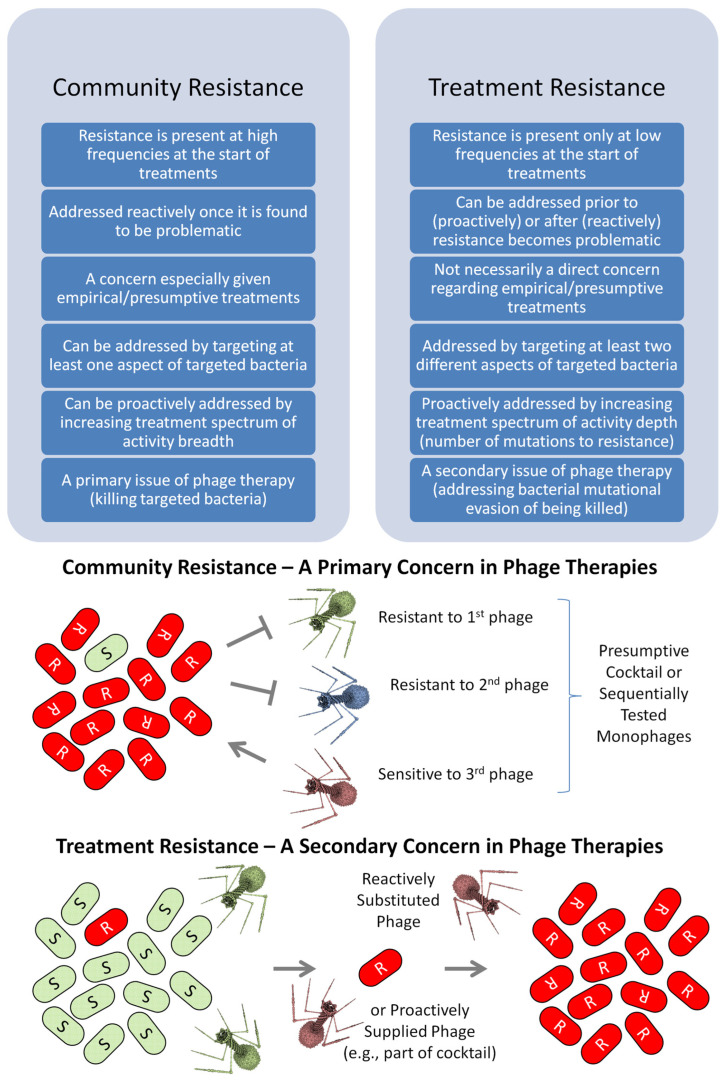
Contrasting community resistance with treatment resistance. Community resistance is bacterial resistance to a phage or phages (red, “R”, bacteria) that has become more or less evolutionarily fixed prior to the start of treatment. This is resistance that had been acquired especially during passage outside of the patient rather than bacterial resistance that develops within a patient during treatment. Successful phage therapies, however, are predicated on applying phages to which a majority of targeted bacteria are susceptible (green, “S”, bacteria). Consequently, there is a consistent requirement to address community resistance to phages at the commencement of phage treatment. Though not all phage treatments seem to be substantially affected by bacterial evolution that can occur during therapies (i.e., ‘treatment resistance’), at least some phage treatments have been found to require phage substitutions in response to reduced phage effectiveness against targeted bacteria. Thus, just as can be the case during antibiotic treatments, the occurrence of treatment resistance is considered to be an important issue by the phage therapy community. How to address treatment resistance, either reactively or proactively, is therefore the primary consideration of this review.

Just as for antibiotics [30], an important issue with phage therapy is bacterial resistance to phages, e.g., [31,32,33,34,35,36,37,38,39,40,41,42]. Also just as with antibiotics, it is possible to distinguish the problem in terms of when it is acquired by bacterial populations; community vs. treatment resistance [38,43] (Figure 1). With community resistance, a majority of the to-be-treated bacteria are resistant at the start of a treatment. Community here is a reference to human populations or their environments within which diverse strains of bacterial pathogens can circulate. These are bacteria that can display dissimilar phage resistance patterns, which thereby may foil the antibacterial activity of at least some treatment phages. Equivalent is the phrasing, ‘community-*derived* resistance’. With treatment resistance, resistance instead is present at only low frequencies at the start of a treatment (see also, e.g., [44,45,46], for the latter’s equivalent usage); equivalently, ‘treatment-*derived* resistance’. Treatment resistance thus is seen only if bacteria are first substantially affected by treatment, something that by definition is not the case unless community resistance is first overcome.

Phage-resistant bacteria usually are present so long as bacterial populations are large enough. These mutationally resistant bacteria, however, do not necessarily always dominate a bacterial population [35,38,39,45]. Resistance thus may be addressed [35,45,47,48] at different stages of bacterial infections. These stages can include the following:

(i) Prior to resistance becoming problematic (lower frequency resistance);
(ii) While resistance develops into a concern (higher frequency resistance);
(iii) Only once resistance is present at a very high frequency.

Community resistance, implying a very high initial frequency of resistance, must be addressed prior to the start of treatment (iii). For treatment resistance, by contrast, it is plausible to delay applying anti-resistance strategies until after a relatively large increase in the frequency of resistance has been identified (ii or iii). It is also possible, however, to attempt to thwart resistance evolution from the beginning of a treatment (i). The latter leads to the possibility of addressing both community and treatment resistance concurrently. Optimization of both anti-community and anti-treatment resistance strategies simultaneously, however, can be more challenging than optimizing each on its own [49].

The distinction between community and treatment resistance can be similarly considered for antibiotics. Thus, from Baym et al. [50], “To deploy the correct strategy against a specific mechanism of resistance, we must be able to differentiate at the point of diagnosis what… an infection is already resistant to [here, community resistance] and the potential it has to develop resistance [here, treatment resistance].” That distinction, though, contrasts with that of Torres-Barceló et al. [45], who instead differentiated between reducing the occurrence of mutation to resistance and minimizing the utility of resistance mutations. Both of those would fall instead under the category of combating treatment resistance—resistance that arises in the course of treatment.

Observation of treatment resistance is often associated with treatment-mediated reductions in the prevalence of treatment-sensitive bacteria. Hence, treatment resistance can be associated, at least transiently, with seeming treatment success, unless grow-back of the resistant bacterial population occurs. That grow-back is the principal medical concern with bacteria evolving phage resistance during treatment. This review therefore examines approaches that can be utilized to combat the evolution of bacterial resistance to phages. These are distinguished into those that either could arise during phage therapy (Section 4) or actually do arise during treatment (Section 3). Indicated in many cases are both the strengths and weaknesses of the varied approaches that may be used to combat this resistance, as can evolve during phage treatment.

## 2. Treatment Resistance

**Figure 2:**
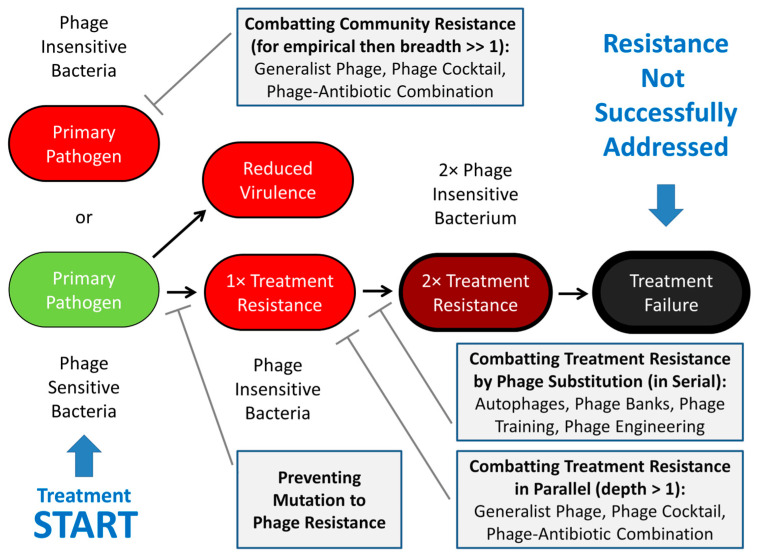
Summary of topics addressed. Bacteria potentially present at the start of treatment are indicated to the left. Progression of infections and treatments goes from left to right. Evolution of resistance to multiple phages, particularly as a consequence of the occurrence of multiple mutations to phage resistance (“2× Treatment Resistance”), need not always occur. This, however, is shown in the figure to give an indication of what both phage substitution and especially in-parallel treatments are also combating, in addition to simply striving to reduce bacterial numbers. To avoid clutter, “Reduced virulence” and “Reduced fitness” of “treatment resistant” bacteria (Section 4.2) are not shown but would be placed under “Combating treatment resistance in parallel”, lower-left. “Breadth” and “Depth” [49] are considered especially in Section 4.3.2 and Section 4.4, along with Section 2.3.

Bacteria can acquire resistance via either mutation or horizontal gene transfer [51,52,53,54,55,56,57]. After phage treatment has been successfully initiated, however, it is especially bacterial mutation to resistance that is a concern. Strategies addressing such treatment resistance can be either in response to its occurrence (Section 3; points ii or iii, above) or instead can act by interfering with its evolution (Section 4; point i, above). The different strategies considered include substitution with phages obtained either as autophages (Section 3.1), from phage banks (Section 3.2), or via phage training (Section 3.3). In addition, phage engineering is mentioned in Section 3.3 but is otherwise not emphasized here. Alternative strategies are those minimizing bacterial mutation to phage resistance (Section 4.1), employing phages targeting bacterial fitness or virulence determinants (Section 4.2), use of phages that recognize multiple bacterial receptors (Section 4.3), employing phage cocktails (as equivalent to ‘polyphages’) (Section 4.4), and combining phages with non-phage antibacterials, particularly antibiotics (Section 4.5). See Figure 2 for a summary.

Many of these approaches can be used together. Their mechanistic commonality—except as considered in Section 4.1—is targeting either serially (Section 3) or simultaneously (i.e., in parallel; Section 4) multiple aspects of the treated bacteria. The goal is to assault more characteristics of individual bacteria than typically may be targeted by single phage types alone, i.e., by a monophage. This is achieved by having treatments “Target different modalities” [58] associated with individual bacteria.

Many of the reviewed strategies are also discussed by McCallin and Oechslin [36], though from different perspectives than as considered here. See also Bleriot et al. [59] for multiple additional considerations regarding increasing phage effectiveness against phage-resistant as well as phage-tolerant bacteria.

### 2.1. Serial vs. Parallel Anti-Resistance Strategies

Serial treatment approaches involve especially “Phage substitution” [36]. Equivalently, Ślopek et al. [60] noted that (p. 570, emphasis added), “In the case of confirmed resistance, bacteriophages were *changed*.” Alternatively, there are in-parallel approaches to combating phage resistance. As contrasting strategies, dissimilar approaches to targeting different modalities thus are either added to treatments one after another—that is, serially, though often with substantial delay prior to substitution (days, weeks, etc.)—or are instead implemented simultaneously (in parallel). If appropriately formulated, in-parallel strategies, however, may instead be used to address community resistance rather than or in addition to combating treatment resistance.

Note that Section 4.1, which addresses the utility of attempting to eliminate the occurrence of bacterial mutations, is omitted from this categorization because it fits neither the serial nor parallel framework. Note also that an alternative meaning of the phrase, ‘phage substitution’, but not intended here, is to incorporate a new phage type into a not necessarily personalized phage product [61]. For discussion of challenges facing more widespread implementation of personalized phage therapy, see [62].

### 2.2. Reactive vs. Proactive Anti-Resistance Strategies

Anti-phage-resistance strategies can also be differentiated into those that are either reactive or proactive (Figure 1). Reactive strategies are implemented only once phage resistance has become a concern (points ii and iii in the Introduction). In contrast are proactive strategies, which are implemented especially at the start of phage treatments (point i).

**Figure 3:**
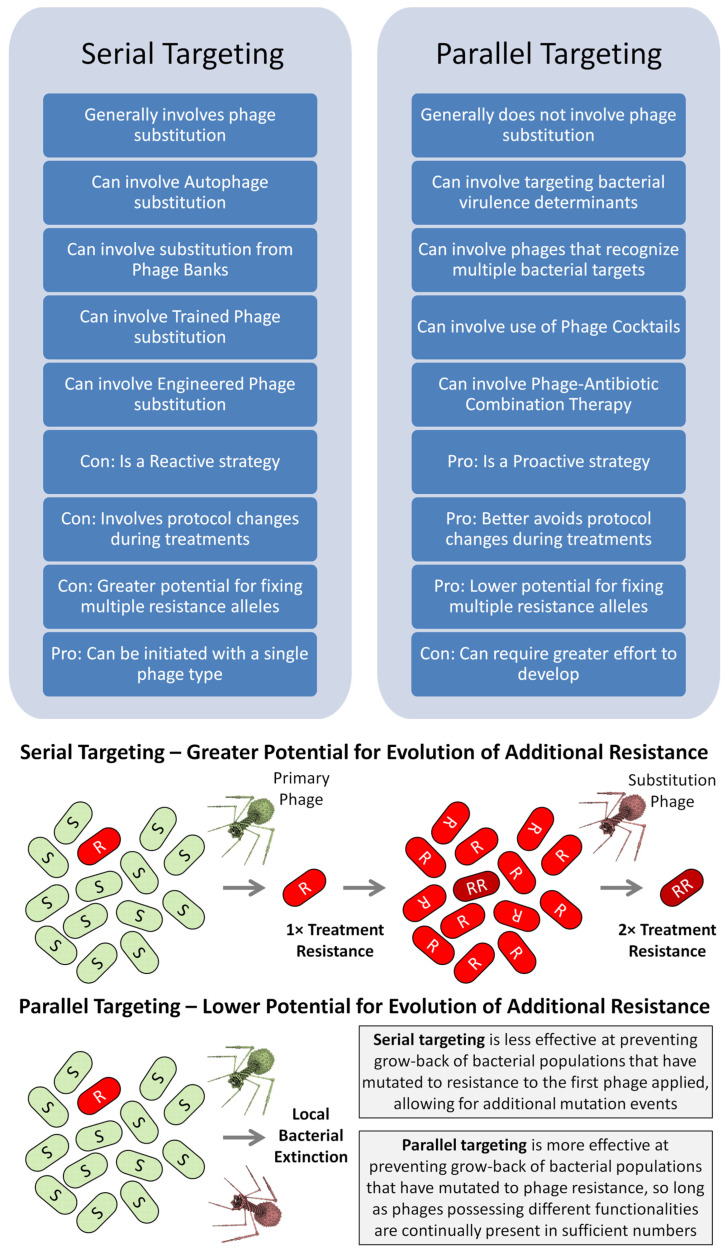
Contrasting the different anti-treatment-resistance strategies. Serial targeting involves ‘phage substitution’ (Section 3). These are reactive interventions that normally would be undertaken only if the occurrence of treatment resistance were identified and especially if treatment resistance were found to be interfering with treatment success. Proactive treatment approaches, by definition, are instead anticipatory and otherwise generally do not involve phage substitutions (Section 4). “Fixing” in the figure refers to evolutionary replacement of an allele within a population, such as replacement across a bacterial population of a wild-type bacterial allele with one that instead confers phage resistance. A theme of this review is that strategies addressing the problem of phage resistance typically will involve targeting more than one aspect of a bacterium. Reactive strategies target different aspects of bacteria at different times, whereas with most proactive strategies, the different targeting is implemented simultaneously.

Reactive strategies, as covered in Section 3, are used predominantly to address treatment resistance rather than to combat community resistance. They consist of phage substitutions and thus of serial phage application. They also tend to be more personalized in their implementation. Proactive strategies, of course, are also suitable for addressing treatment resistance, as is emphasized in Section 4. Those strategies instead tend to be implemented in parallel. They also tend to represent strategies that, as currently envisioned, have been worked out well in advance. They therefore usually are ready-made rather than personalized approaches, i.e., prêt-à-porter [63] (also recently described as “Fixed” [20]). See Figure 3 for a summary.

### 2.3. Breadth vs. Depth of Activity

To be effective, most anti-treatment-resistance approaches require sufficient depth of antibacterial activity. This describes, essentially, the number of distinct modalities targeted on individual bacteria. For example, this can be and often is the targeting of different bacterial surface receptor molecules displayed by the same bacterium. To effectively address community resistance, especially presumptively/empirically, treatments instead must possess sufficient breadth of activity.

Breadth also involves targeting different bacterial modalities. In this case, however, targets are not necessarily associated with a single bacterium. That is, A and B as targets need not be associated with any one bacterium, as is required instead for activity depth, but must be spread across multiple possible target bacterial strains. Simultaneously addressing both treatment and community resistance, especially empirically, requires in turn sufficient *breadth of depth* of activity (Section 4.4.3).

## 3. Reactively Addressing Treatment Resistance

This section overviews three approaches to combating treatment resistance, all involving phage substitution. Each can be viewed not so much as serving to minimize bacterial evolution of resistance as instead responding to its occurrence. They are thus the phage equivalent of switching antibiotics after a patient’s infection comes to demonstrate antibiotic resistance. In addition, without ongoing competition from phage-sensitive bacteria, phage-resistant bacteria may undergo some degree of grow-back prior to the application of a new agent, during which more bacterial mutation to resistance can occur. That is, from Rohde et al. [35], giving “bacteria the opportunity to develop resistance against one active phage at the time.” Phage substitution thus is inefficient in terms of battling further resistance evolution and this is because it is a reactive rather than proactive approach.

Phage substitution is often also a ‘sur-mesure’ treatment [63]. This literally means “on measure”, though idiomatically the meaning is closer to “custom-made”. In the case of autophages, as considered first, it instead is more like “custom-isolated”. In addition to supplying substitution phages, the various approaches considered in this section may also be used to provide primary phages toward addressing instead community resistance. In all cases, prior to their use, phages should be characterized to avoid phages that are capable of displaying lysogenic cycles [64,65] or which encode bacterial virulence factors such as toxin genes [66,67,68]. Those are two properties that make phages unsuitable for use as therapeutics [68].

### 3.1. Autophages

Autophages are bacterial viruses that have been isolated using a bacterial strain, as the phage isolation host, that was obtained from a to-be-phage-treated patient. Alternatively, or in addition, these are phages that have been isolated from the same environment as the isolation host, including from a patient’s own body [69]. The focus here is on the first definition: phages isolated from some environment ‘against’ a specific bacterial etiology. See Appendix A for an historical look at the distinction. See Table 1 for a summary of the pros and cons associated with this approach.

#### 3.1.1. Explicit Avoidance of Cross-Resistance

In substituting for an original treatment phage, the bacterial strain being treated must not display cross-resistance [49,70,71,72,73,74,75]. That is, in the course of having become resistant to the original treatment phage, they must not have also become resistant to a subsequently applied phage. That concern of cross-resistance can be avoided, however, if the mutated, now phage-resistant bacterial etiology is used as the new isolation host [76,77,78], i.e., to generate an autophage. Autophages, in other words and by definition, are able to successfully infect the bacterial isolation host whether or not that host has previously acquired resistance to other treatment phages.

Use of autophages, or indeed use of any new phage isolate, nonetheless can be prohibitive to the extent that phage characterization beyond host range determination is required prior to their therapeutic use. The process of newly isolating phages also is relatively time-consuming—“a few days to weeks” [63]—and requires expertise in both phage isolation and the noted phage characterization.

#### 3.1.2. Related Terms

Contrasting the strict definition for autophage used above, Zaldastanishvili et al. [37] describe a “custom phage” as “an individualized phage preparation”. This presumably is an example of personalized medicine rather than specifically a phage that has been isolated against a to-be-targeted bacterial etiology. The same group in addition describes these as “tailored bacteriophages” which “are targeted at specific strains that have been isolated and identified in patients’ biological samples.” That definition, though, also is not necessarily of autophages, but instead could include phages obtained from a phage bank (Section 3.2). Similarly, see [79,80] for equivalent concepts of “Custom phage” and “Custom-made bacteriophage therapy”.

Oechslin [34] describes this presumed autophage/phage bank approach as, “The personalized phage strategy uses single phages or targeted phage cocktails directly formulated from a phage bank according to the pathogen isolated from the patient.” Thus, an autophage as emphasized here should be viewed as a specific kind of custom, custom-made, or tailored bacteriophage; one that specifically has been *isolated* “according to the pathogen isolated from the patient.”

### 3.2. Phage Banks

If a phage collection already exists, that collection can be described as a phage bank [20,81,82,83,84,85,86,87], PhageBank [88,89,90], phage library [91], phage biobank [92], or simply the noted phage collection [31]. If those phages have been previously characterized [91,93,94], then substitution of one treatment phage for another can be a relatively rapid exercise. Alternatively, it is possible to use a phage bank to substitute in an entirely new phage cocktail [33]. For clinicians lacking direct access to phage banks, it is also possible to obtain phages via “crowdsourcing”. This is the supplying of phages by otherwise independent researchers to interested physicians [95,96], though presumably these would tend to be phages of varied prior characterization. Phage banks, and their use in phage substitution, have been a characteristic especially of phage therapy as practiced in Poland [31]. See Table 2 for a summary of pros and cons associated with the use of phage banks to supply substitution phages.

#### 3.2.1. Maintaining an Optimal Phage Bank

Though phage banks can offer speed of access to substitution phages, the literal costs of phage banks are their creation, maintenance, and operation. The banked phages have to come from somewhere. They ought to be characterized prior to when they are needed, to speed up subsequent implementation. Banked phages also have to be stored, preserved, and amplified for use [94].

The latter requirement means that a collection of phage-amplifying bacterial hosts must also be stored. Ideally, these host bacteria will have been characterized, such as for the presence of resident prophages [35,93,94]. Amplification protocols need to be optimized on a per-phage basis to achieve adequate phage concentrations for clinical use. More recently there have been calls for cell-free phage production [97,98]. That approach, however, does not seem to be mature nor easily implemented, given the small number of phages to which it appears to have been so far applied.

For more routine use, phage banks likely will also need to employ Good Manufacturing Practice, i.e., GMP [99]. Banked phages in addition have to be tested against specific bacterial etiologies as required. Phages ideally also will be properly purified [94] as well as titered prior to being appropriately transported to users. These requirements can be the case for autophages as well.

#### 3.2.2. Still Time Lags but Fewer Time Lags

While it is possible for institutions or even wide geographical areas to share in the costs of having access to phage banks, or for phages to be generated locally on demand [100,101], there inevitably will be a time lag that cannot be easily overcome between recognition of a need for specific new phages and clinical access to those phages. For instance, these can be time lags resulting from phage host range testing (to match specific etiologies) and from phage amplification as well as due to transportation; the latter is particularly an issue if a phage bank is not found nearby or on site. These time lags, though, will not be as great as those present if new phages have to be isolated (e.g., autophages; Section 3.1) or if new phages have to be generated by other means (e.g., phage training; Section 3.3). Phage banks thus can be viewed as an optimized approach to sur-mesure phage therapy. That optimization is seen particularly in terms of reducing some of the delays incurred prior to physicians accessing these personalized phage preparations, though without eliminating those delays entirely.

### 3.3. Phage Training

An alternative to substituting in an already existing or newly isolated phage is phage training; e.g., [45,48,58,90,102,103,104]. This also can be described as phage adaptation, phage evolutionary training, in vitro evolution, phage pre-adaptation, phage breeding, or phage laboratory evolution [35,39,40,42,105,106,107,108,109,110]. It involves directed evolution [111] via artificial selection, especially selection for spontaneously occurring phage mutants. The goal of these processes often is to modify phage host ranges to obtain or to improve upon a phage’s ability to lytically infect otherwise fully or partially phage-resistant bacteria. The approach, however, takes both time and phage handling expertise [32,35,112,113]. Though phage training as well as autophages can successfully treat bacterial infections caused by specific bacterial strains, the same phages may not display similar therapeutic effectiveness against less closely related bacterial strains [39]. See Table 3 for a summary of pros and cons associated with phage training.

#### 3.3.1. Advantages of Training Phages

Phage training can have a characterization advantage over autophages if phages are modified through mutation alone or instead due to epigenetic changes [114,115,116,117]. This is because many properties of trained phages should remain similar, besides host range, to the properties of phages from which they have been derived. For instance, a single phage mutation may result in modification of only a phage’s receptor binding protein [118,119]. Characterization of so-modified phages consequently should take less time and effort [35]. Phage-encoded bacterial toxin genes, for example, should not be able to evolve de novo over the short term by mutational or epigenetic phage modification alone. Nor should phages be able to rapidly evolve an ability to display lysogenic cycles, unless a treatment phage has been mutationally derived from a previously temperate phage. Toxicity testing therefore should be less necessary than for completely new phages. Whole-genome phage sequencing nonetheless should still be undertaken, if only to document that only minimal phage evolution has occurred.

#### 3.3.2. Disadvantages of Training Treatment Phages

Potentially problematic are the immunological characteristics of trained substitution phages, if they have been only minimally genetically modified from original treatment phages. These especially serological characteristics [120], in other words, should remain somewhat consistent with those of their parental phages, thereby likely retaining an ability to re-stimulate a patient’s immune system. The resulting humoral response may negatively impact treatment success [121,122], though not necessarily always [123,124]. Nonetheless, serological familiarity to the body presumably should not be viewed as advantageous in a treatment phage.

Alternatively, phages that are new to a patient may also be trained—phages for which the body is potentially or even likely immunologically naïve. These new phages would need to be much more thoroughly characterized, however, whether or not they are subsequently trained. For to-be-trained phages derived from phage banks, such characterization ideally will already have taken place. Training already characterized phages, ones that have not yet been therapeutically applied to a patient, thus could represent an ideal means of overcoming limitations to phage bank collections. This is in contrast to the isolation of autophages or training phages that had previously been used on the same patient.

#### 3.3.3. More than Just Mutational Change

A more powerful approach to phage training, more than simply selecting for spontaneously occurring phage host range mutants, is a procedure often referred to as that of Appelmans [107,109,111,125,126,127,128,129,130], and see also [131] as well as [132]. With this approach, recombination between different phages can also take place. The resulting phages, however, can be somewhat more modified genetically than trained phages that have changed only through point mutation or epigenetically. Trained phages that have been recombinationally modified, especially following recombination with uncharacterized or poorly characterized prophages, thus should be subject to thorough characterization prior to therapeutic use. At the same time, these recombinant phages, if partly derived from previously used treatment phages—and thereby sharing virion proteins—could still possess an ability to further stimulate a patient’s immune system. The power of recombination as a contributor to viral evolution, particularly in nature, has been reviewed more generally by Bono et al. [47].

More controlled recombinogenic genetic modification can be accomplished via phage genetic engineering [19,39,48,59,133,134,135,136,137,138,139]. The most prominent example of phage engineering for phage therapy, however, was used to convert temperate phages to ones which instead were obligately lytic, one of which was then more traditionally trained to improve its infection characteristics [64]. This was carried out for the sake of combating community resistance rather than treatment resistance.

Note that there is no guarantee that these products of recombination will be highly effective, or even just useful therapeutically, other than displaying whatever property has been selected for, which in most cases is a new host range. The same therapeutic usefulness caveat, however, is also valid for autophages and phages derived from phage banks. Phage engineering as well as phage training, Appelmans’ technique, use of phage banks, or isolation of autophages all also represent reactive treatment strategies, if implemented only after phage resistance has become problematic. For a variety of reasons, addressing the problem of treatment resistance proactively instead can be preferable, as the next section reviews.

## 4. Proactively Addressing Treatment Resistance

Treatments involving serial phage substitutions are inherently more complex than strategies without substitutions. This is because substitutions, at a minimum, require at least two temporally spaced introductions of new phages [35]. Since new phages would tend to be substituted particularly as previous phages became ineffective, serial phage treatments may also take longer to complete. There should therefore be some utility to preventing rather than reacting to bacterial evolution of resistance—making treatments proactive, as reviewed in this section. Mostly these approaches consist of parallel targeting—“a and b”, rather than the “a then b” of serial applications. First considered, though, is the strategy of simply preventing bacterial populations from growing large in size, to reduce bacterial mutation to resistance. Note in any case that avoiding substituting new phages into therapies is only possible if initial treatments are therapeutically successful by themselves.

### 4.1. Minimizing the Occurrence of Mutation to Resistance

A proposed general approach to preventing resistance evolution is to kill bacteria both fast enough and soon enough that they are unable to reach population sizes large enough that mutation to phage resistance can readily occur [45]. That is, from Oromi-Bosch et al. [39], “The straightforward approach to overcome bacterial resistance to phages is to design a phage therapy that maximizes the rate of phage killing across genotypes of target clinical isolates, thus restricting bacteria from growing to large population sizes.” Such an approach would have to overcome bacterial mechanisms of tolerance to phages [38], which could interfere with rapid bacterial killing. Another limitation is that for such a strategy to effectively prevent the evolution of resistance, a bacterial infection would have to present clinically prior to mutation to resistance. Sufficiently early detection, however, is mostly clinically unlikely. Nonetheless, this section probes this concept of seeking to minimize bacterial mutation to phage resistance further, as it is not entirely inapplicable. See Table 4 for a summary of its pros and cons.

#### 4.1.1. An Advantage of Prophylactic Treatment

Preventing bacterial populations from growing large enough to mutate to phage resistance could be viewed as a secondary benefit of prophylactic phage therapy. Such treatments ideally act prior to successful bacterial colonization, let alone prior to bacterial replication to high numbers. Beyond prophylaxis, simply replacing phages soon enough (Section 3) might catch bacteria before they reach population sizes such that new mutations to resistance to substitution phages have occurred. That scenario, though, would seem unlikely. This is due both to delays in finding new phages (see again Section 3) and the potential for bacterial populations to regrow rapidly to large sizes. Providing these new treatments with sufficient rapidity, however, is a primary advantage of using ‘combination’ therapies (Section 4.4 and Section 4.5 but also Section 4.3). That is, those approaches, including as considered in Section 4.2, may be viewed as a kind of treatment resistance prophylaxis: they address resistance evolution prior to its becoming problematic rather than explicitly prior to mutation to resistance.

#### 4.1.2. Less Applicable to Treatment of Established Bacterial Infections

This idea of preferably treating bacterial infections with phages earlier rather than later can be traced back at least to d’Hérelle [140]; see [141] for the relevant quotation from that 1930 book translation. Indeed, particularly problematic for phage therapies, though also very common, is the treatment of chronic, long-standing, or persistent bacterial infections [142]. These infections for the most part are no longer substantially growing in terms of numbers of bacteria present. As a consequence, preventing mutation accumulation through limiting bacterial growth to large numbers may no longer be possible. It is for these various reasons that this approach of “Minimizing bacterial resistance” [39], as discussed in this section, is not considered further in this review.

#### 4.1.3. Monitoring Treatment Resistance

There exists a related issue to minimizing bacterial mutation to phage resistance: that the presence of resistance mutations alone should not be viewed as an indication of problematic treatment resistance. Rather, just as the presence of bacterial mutations to phage resistance should not be interpreted as an indication of treatment success (see the last paragraph of the Introduction), mutations to resistance should not necessarily be seen as an indication of treatment failure, as this section considers.

**More to evolution than mutation.** The concept of evolution has many facets, mutation being only one [143]. Thus, from Rohde et al. [35]—though addressing community resistance rather than treatment resistance (emphasis added)—“The phage resistance problem is not caused by the de novo emergence of phage resistant clones, but by the *selection* of naturally present phage resistant isolates…” While as noted this observation was made in the context of community rather than treatment resistance, the underlying principle still applies: given the importance of selection, it can be somewhat meaningless for studies to just indicate that bacterial resistance to phages is present “during” treatments. This generally is because, if genetically and ecologically possible, then resistance to one or more phages in most cases is expected to have evolved prior to the start of treatments; that is, again, unless those treatments have been initiated very early. Bacterial mutations to phage resistance, in other words, occur spontaneously rather than in response to phage treatment, as indicated in the classic fluctuation test study of Luria and Delbrück [144].

Mutations, consequently, are expected to be present so long as a bacterial population is large enough in size. Simply showing that phage-resistant bacteria are present within a bacterial population therefore should not alone be viewed as an indication of phage therapy ineffectiveness, nor of selection for phage-resistant bacteria.

**More to treatment failure than increased frequency of resistance.** A more pertinent question regarding treatment resistance could be, have resistance mutations increased in frequency over the course of phage therapies [145]? That is, increased in frequency as due to the selective pressures provided by phages, since changes in mutation (allele) frequencies also represent a form of evolution during treatments. Even observation of increases in the frequency of phage-resistance alleles within a bacterial population, however, should not be viewed as a sufficient indicator of problematic treatment resistance, as the following paragraph considers.

**Quantity rather than frequency usually is key.** In terms of judging treatment efficacy, instead it is important to show as well that the number of phage-resistant mutants has increased following phage application. This is because reducing the frequency of phage-sensitive bacteria will automatically increase the frequency of phage-resistant bacteria, without necessarily also increasing the latter’s clinical impact. Increases in frequency, in other words, can be due to changes in the denominator of frequency calculations (decrease in the number of bacteria) rather than due solely to changes in the numerator (increase in the number of phage-resistant bacteria). This emphasis on numbers is important since, as noted, the presence of phage-resistant mutants is to be expected within most bacterial populations, so long as the bacterial population is large enough. Treatment resistance is nonetheless defined here as an increase in resistance frequency that occurs in the course of treatments, though as noted, that should not be viewed in and of itself as an indication of treatment failure.

Oechslin [34] makes this latter point by noting—in this case for phage-resistant bacteria that display altered virulence factors (“Variants”)—that (emphasis added):

The question then arises as to whether these variants were mere innocuous bystanders on the way of being eliminated by host defences, or whether they could still produce infection. In any case, the ideal experimental setting should be to apply the Koch postulate [*sic*] and inoculate the variants to the animals in order to re-evaluate their infectivity. Indeed, *recovering phage-resistant variants from in vivo samples may not be automatically synonymous with therapeutic failure, a counter-intuitive concept that appears to apply to phage therapy.*

**Monitoring changes requires pre-treatment testing.** Even more fundamental is this point made by McCallin and Oechslin [36], p. 82: “Without both pre- and post-phage sensitivity testing, it is not possible to ascertain if resistance develops throughout the course of treatment”.

These difficulties in preventing the occurrence of bacterial resistance to phages, or in ascertaining whether treatment resistance is clinically relevant given its occurrence, point to a utility of addressing treatment resistance prior to its (potentially) turning into a problem. That is, by essentially prophylactically limiting the ability of newly formed phage-resistant bacterial mutants to proliferate during treatments.

### 4.2. Targeting Bacterial Fitness/Virulence Determinants

There are at least three consequences of phage treatment that could contribute to phage therapy success: (1) phage-mediated reductions in bacterial numbers, (2) phage-mediated dispersion of bacterial biofilms, and (3) changes to prevailing bacterial genotypes during the evolution of phage resistance. Reducing bacterial numbers has obvious direct therapeutic utility. It may also help to give immune systems an upper hand over bacterial infections, perhaps particularly given patient immunocompetence [40]. Phage-mediated biofilm dispersion is likely similarly helpful including potentially increasing antibiotic impacts [146,147], and perhaps also vice versa [148]. Such indirect effects, however, may be difficult to prove since phage treatments ideally also kill biofilm bacteria [149] and/or degrade extracellular polymers [150]. The emphasis in this section, though, is on point (3): that mutation to phage resistance can weaken bacteria in terms of their ability to resist non-phage aspects of bodies and treatments. See Table 5 for a summary of pros and cons associated with this targeting of bacterial fitness and virulence determinants.

#### 4.2.1. Collateral Sensitivity and Antagonistic Pleiotropy

Chan et al. [151,152] used phage OMKO1 for human therapy, which targets as its adsorption receptor a bacterial efflux pump. This phage was used explicitly to reduce the potential of phage-resistant bacteria to also display antibiotic resistance. The strategy relies on phage resistance occurring through genetic deletion or modification of the efflux pump protein. Mutations adversely affect efflux pump suitability as a phage receptor, however, do not always work out so favorably from a phage therapy perspective [153]. See also Kim et al. [154], who found that phage OMKO1 requires intact *Pseudomonas* flagella as well for successful adsorption.

The Chan et al. [151,152] OMKO1 story can be described as an example of collateral sensitivity [1,155,156]. This is where resistance to one selective agent increases sensitivity to a different selective agent. More generally, this is where a single mutation leads to both detrimental and beneficial outcomes, in this case for the bacterium. The same type of scenario can also be described as negative cross-resistance [50], antagonistic pleiotropy [75,153,157], or simply a tradeoff [158]. However, these latter terms—antagonistic pleiotropy and tradeoff or simply “pleiotropic effects” [40]—are more broadly applicable than just to phage and antibiotic resistance.

Note also the opposite concept: synergistic pleiotropy. Here, mutation to phage resistance also results in increased antibiotic resistance [39,75], which of course could be undesirable for phage–antibiotic combination therapies (Section 4.5). Synergistic pleiotropy, though, is basically cross-resistance [49], i.e., one mutation—two resistances. That in turn contrasts with the concept of negative cross-resistance, as considered in the previous paragraph. Related to this issue, though not involving pleiotropy, is an observation that exposure of *Pseudomonas fluorescens* to sub-inhibitory concentrations of streptomycin can result in evolution of both reduced streptomycin and phage sensitivity, in that case potentially due to the streptomycin serving as a mutagen [159].

#### 4.2.2. Reciprocal Collateral Sensitivity

Collateral sensitivity at its most powerful works in both directions: resistance to one agent results in increased sensitivity to another, and vice versa. With the Chan et al. [151,152] example, collateral sensitivity indeed occurred in both directions, with phage resistance (loss of efflux pump) resulting in increased antibiotic sensitivity, while antibiotic resistance (gain of efflux pump) resulted in increased phage sensitivity. This can be described as a *reciprocal* collateral sensitivity [50,155,160]: resistance to A increases sensitivity to B and resistance to B increases sensitivity to A. This contrasts with simple collateral sensitivity (Section 4.2.1), i.e., where resistance to A increases sensitivity to B but resistance to B does not necessarily increase sensitivity to A.

Achieving reciprocal collateral sensitivity among only phages, rather than the above phage–antibiotic example, can involve bacterial capsule gain vs. loss. This works for phage pairs in which one requires bacterial capsule for adsorption, while the other requires a lack of a bacterial capsule for adsorption [161,162]. Acton et al. [1] instead identified phages for which the inactivation of genes involved in LPS (lipopolysaccharide) synthesis resulted in resistance to one phage (SPLA1a) while the same mutations conferred sensitivity to another (SPLA5b). Mu et al. [156] showed both effects—with and without a capsule and with and without modified LPS—though with a somewhat more complex evolutionary trajectory.

What could be especially interesting would be mutations with opposing phenotypic impacts but with both involving loss of function, which typically is easier to achieve than gains in function. This is rather than a loss of function in one direction (e.g., a loss of efflux pump, capsule, or LPS-modifying enzymes) but gain of function in the other (such as gain of the same). Examples of such mutual loss-of-function reciprocal collateral sensitivity, involving resistance evolution to phages, however, may be elusive.

#### 4.2.3. Phage Targeting of Bacterial Virulence Factors

Though novel, the approach of Chan et al. [151,152], and collateral sensitivity more generally, still represents a variation on the idea of using phages that target bacterial virulence factors. The goal is to reduce the virulence of phage-resistant bacteria, e.g., [39,71,76,90,163,164,165,166,167,168,169] or to increase the sensitivity of bacteria to antibiotics [39,75,151,153,170,171,172,173,174,175,176,177,178]; greater resistance to antibiotics also can be associated with increased sensitivity to phages [179,180]. These results, in turn, are variations on the idea that the fitness of resistant bacteria often is lower than that of isogenic, in this case, phage-sensitive bacteria [158,167,181,182,183,184,185]. Note though, from [36], p. 75, that “Assays in rich media might not reflect their true clinical viability” of phage-resistant bacteria.

These effects as noted are examples of antagonistic pleiotropies, e.g., [38,157], which can also be described as evolutionary [50] or fitness [185] tradeoffs. In their classic, 1982 phage therapy study, Smith and Huggins [163] took advantage of such a tradeoff, with phage resistance resulting in increased immune system sensitivity. Lenski and Levin [186] described this as an Achilles’ heel strategy. More recently, this has been called phage steering [39,187,188] as well as directing phage resistance evolution [45]. McCallin and Oechslin [36], however, instead use the heading “Resistance reversion” to describe the same concept (p. 81); see also [189]. In addition to pleiotropic effects, phage resistance can lead to losses by bacteria of whole antibiotic resistance genes [161,190] as well as, not surprisingly, to the loss of other fitness- and/or virulence-relevant genes [191].

In all of these cases, the explicit concept is that bacteria that evolve phage resistance can be less able to cause or continue to cause disease [51]. Therefore, therapeutically targeted bacteria may either (1) be eliminated directly by phage action or, instead, (2) may be eliminated indirectly because phage-resistant bacteria are less able to continue to infect a patient, as seen by Chan et al. [151,152]. This in any case is the targeting of at least two aspects of a bacterium: their viability and their virulence. Both result in an increased potential for the body to clear infections despite the potential for bacteria to mutate to phage resistance.

#### 4.2.4. Limitations to Collateral Sensitivity

This approach of using phages therapeutically that are able to target virulence-affecting bacterial features is both a legitimate utility of phage therapy and potentially equal to phage cocktails (Section 4.4) in its ability to combat phage resistance. Claims of its equivalence to the utility of phage cocktails, though, come with multiple caveats. First, to rationally develop this virulence-reducing approach, it is important to prove that phage-resistant bacterial mutants are not only less virulent but also, for prêt-à-porter use [63], that this tendency will be true for most or all bacterial strains that might be targeted [178]. Second, the success of this approach may depend on the state of a patient’s immune system as immunocompromised individuals may be less able to clear infections even if consisting of lower-virulence bacteria [168,192]. Third, this approach can offer little advantage for empirical treatments to address community resistance, unless by chance a phage with this property happens to target the infecting bacterium.

Whether as monophages or as polyphages, using phages for which resistant bacteria display reduced virulence nevertheless may be viewed as largely advantageous. This, though, comes with an additional caveat. Namely that this property would be less advantageous the less consistently mutations to resistance, by a given targeted bacterium, indeed result in reductions in bacterial virulence [155]. That is, 50% of resistance mutations rather than 100%? These odds, though, should be improvable if it is possible to hit targeted bacteria with two or more phages for which resistance mutations give rise to lower virulence, along with minimal cross-resistance.

### 4.3. Individual Phages Recognizing Multiple Receptors

As discussed in the previous section, Section 4.2, phages that target bacterial virulence aspects can impact bacteria in two ways: killing phage-sensitive bacteria and rendering phage-resistant bacteria less capable of causing or continuing to cause disease. These phages, however, directly impact targeted bacteria in only one manner—killing phage-sensitive bacteria. Reduced virulence instead is an indirect consequence of phage actions, one resulting from eliminating phage-sensitive bacterial genotypes rather than phages having directly acted upon resistant bacteria or their ancestors [144]. Reductions in virulence are also less easily assessed than the killing of bacteria. By contrast, it is possible for single phage types to directly impact bacteria in two distinct ways. This effect is most easily visualized with phages that recognize more than one bacterial receptor for adsorption. This, for example, is seen with coliphage T2 [58,193] (see also [119,194,195,196,197]). Specifically, with phage T2 knocking out one receptor does not necessarily result in full resistance to the phage. That contrasts with phages requiring both of two different receptors for successful virion adsorption, where the loss of either receptor results in full phage resistance, e.g., [153]. That is, the loss of receptors A and B to achieve full resistance to phage T2 rather than the loss of either receptor A or receptor B to achieve the same full resistance [154,198]. See Table 6 for a summary of pros and cons associated with using such ‘A and B’ phages to combat treatment resistance.

#### 4.3.1. Targeted Bacteria Must Display Both Receptors

Borin et al. [58] described ‘loss of receptors A and B’ phages, such as seen with phage T2, as “Dual-receptor generalists”, while Rojero et al. [199] perhaps would include this characteristic among their criteria for phage hyper-aggression. See also [200] for an evolved phage λ that is able to adsorb using either the LamB (original) or OmpF (new) receptor. Alternatively, individual virions can vary, via a genetic program, in what receptors they use for adsorption. Historically, this strategy has been most prominently associated with coliphage Mu [201,202]. The result of either strategy, T2-like or Mu-like, is that one phage type can do the work of two phage types as ideally two independent bacterial mutations will be necessary to achieve complete resistance to that single phage type. This though is only in principle.

Specifically, for one phage type to do the work of two in reducing bacterial resistance evolution, then both targeted receptors must be consistently found on the same bacterium. If that is not the case, as is classically true for phage Mu, then even though a phage may be able to recognize more than one receptor, it still requires only a single mutation for a specific bacterium to evolve resistance. This issue is not limited to individual phages recognizing more than one receptor, however. Even if one has two different phages, each recognizing a different receptor, these phages will interfere with bacterial evolution of phage resistance only if both receptors are found on the same bacterium. Indeed, as with any phage for successful phage therapy, the targeted bacterium has to be found within the treatment phage’s host range.

#### 4.3.2. Monophage Depth and Breadth

More generally, two antibacterial agents will only be able to combat treatment resistance if they can both impact the same bacterium and not occupy the same cross-resistance group. Elsewhere we have described this property as one of possessing “Depth” of activity [49] (Section 2.3 and Section 4.4). Thus, a single phage type (monophage) that can directly impact a bacterium in two non-identical ways can be said to have a greater depth of activity against that bacterium than a phage lacking this ability. This is true whether the lack of ability is due to an absence of redundancy (recognizes only a single receptor) or instead is due to not possessing redundancy when targeting a given bacterial strain (recognizes only one receptor displayed by a given bacterium despite being able to recognize more than one receptor). Borin et al. [58] point out, however, that even for phages that can recognize different receptors, if an additional, single host molecule is required for successful infection, or simply required for successful bacterial killing, then even dual-receptor recognition may not be sufficient to avoid facile one-step host mutation to dual resistance.

Notwithstanding the latter caveat, having single phages that recognize two different receptors can still be useful even if those receptors are not displayed on the same bacterium. This is especially true when T2-like (or Mu-like) dual-receptor recognition broadens the number of bacterial strains affected, i.e., greater breadth of activity. Such broadening is useful for increasing the potential for successful empirical treatment, i.e., to combat community resistance. Furthermore, this latter utility will remain even if a targeted bacterium can easily mutate to resistance since, as noted, addressing community resistance and successfully preventing treatment resistance are not identical goals. Specifically, overcoming community resistance is about killing what bacteria are originally present while overcoming treatment resistance is about hindering bacterial mutants that could become present.

Additionally, having T2- (or Mu)-like dual-receptor recognition in a single phage may simplify treatments by requiring inundative phage titers [203] for only one phage type (monophage therapy) rather than for two different host range variants (polyphage therapy) [58]. Such two-receptor-targeting monophage therapy would also eliminate any potential for antagonism between different phage types [204]. In short, phages that are dual-receptor generalists should be quite valuable toward addressing both treatment and community resistance, including as components of phage cocktails.

### 4.4. Phage Cocktails

Phage cocktails, also described as polyphages, are combinations of different types of phages that are dosed simultaneously; that is, applied in parallel [29,39,41,48,49,82,105,154,205,206,207,208,209,210,211,212]. This simultaneous dosing is performed to increase some desirable therapeutic property that is less readily achieved using monophages. This is either [213,214] an overall spectrum of activity breadth [47,49,209,215,216,217]—the number of bacterial types that are impacted by a phage formulation—or instead a reduction in the potential for bacteria to evolve to resist phage treatments. The latter can be described as a function of a cocktail’s depth of activity [49] (Section 2.3). Though employing polyphages can be advantageous relative to monophages, phage cocktails are not without limitations [206]. Those include greater development and production costs along with increased complexity. Additionally, different phages may interfere with each other’s infection activities upon coinfection of the same bacterium, i.e., phage antagonism [204]. This section primarily emphasizes using phage cocktails to combat treatment resistance development, which requires cocktail depths of activity greater than 1. This section also distinguishes cocktails designed to combat treatment resistance from cocktails designed to combat community resistance, based on component phage properties. See Table 7 for a summary of pros and cons associated with cocktail use.

#### 4.4.1. Differentiating Breadth and Depth

Often spectrum of activity breadth and spectrum of activity depth are not adequately distinguished in the literature, with the word cocktail used to describe mixtures of phages employed to address either issue. This is unfortunate because to optimize either of these goals, a cocktail may need to possess non-identical properties [43,49]. For instance, to extend spectrum of activity breadth, a cocktail need impact each targeted bacterium with only a single phage. Consequently, to address community resistance empirically using cocktails, there can be utility to employing phages whose host ranges do not overlap. Indeed, a lack of host range overlap can be preferable given a therapeutic goal of greater cocktail breadth of activity.

By contrast, to increase depth of activity, two or more phages must be present in a cocktail, both of which infect the targeted bacterium. Further, the targeted bacterium must not easily mutate to cross-resistance. This is just as two different receptors recognized by a single phage type must both be found on the same bacterium to impact that bacterium’s potential to evolve phage resistance (Section 4.3). At an extreme, for example, there is no utility in combating treatment resistance by employing cocktails of phages whose host ranges do not overlap; though see Section 4.4.2 for an interesting exception to that claim, as well as Section 4.4.3.

In terms of assuring the equivalent anti-treatment-resistance activity, Wright et al. [218] called for employing phages displaying sufficient “Functional diversity”. This they defined as “the constituent phages targeted multiple distinct adsorption receptors.” Greater functional diversity among cocktail phages [218] and greater cocktail depth of activity [49], however, can be viewed as essentially synonymous concepts. Both, that is, can serve as means of quantifying a phage cocktail’s potential anti-treatment-resistance effectiveness. Of historical note, those two articles [49,218], were accepted for publication within eight days of each other. Though published later, see equivalently Borin et al.’s [58] reference of “Different modalities” as well as Rotman et al. [90], who described “Selecting phages that depend on diverse host factors for infection” and that “Successful phage cocktails necessitate multiple phages that depend on different host factors for phage infection to minimize the likelihood that a single mutant can arise against multiples viruses.” Achieving such diversity or depth of activity operationally thus implies that resistance to two or more phages by a single bacterium requires two or more bacterial mutations, thereby curtailing cross-resistance [49].

To optimize cocktail breadth of activity, it thus is useful to minimize phage host range overlap, while to optimize cocktail depth of activity, then phage host range overlap is essential, though not to a point where cross-resistance is likely. Another way of making these points is that while increasing cocktail depth of activity can potentially also increase anti-community resistance—“Functionally diverse phage combinations are likely to be able to target a broader diversity of bacterial genotypes” [218] (and see also [219])—increasing cocktail breadth of activity may not increase anti-treatment resistance at all. Breadth, that is, can be accomplished by adding phages that recognize new bacteria while not necessarily also infecting already cocktail-recognized bacteria, as would result in the opposite of achieving greater depth of activity.

In terms of anti-treatment-resistance activity using cocktails, it is also important that two or more phage types be able to physically reach targeted bacteria at sufficiently high titers in situ [206]. Ideally this is achieved by reaching inundative titers [203], and doing so locally [220], so that individual bacteria are substantially impacted by the different phage types [58,206]. Alternatively, to combat community resistance, then the primary goal need be for only one effective phage to reach each individual targeted bacterium. Combating community resistance thus, again, is about reaching and then killing problematic bacteria whereas combating treatment resistance is about reaching and then hindering the development of problematic resistance, two concepts which are not identical.

#### 4.4.2. Proactive Autophages

There exists an exception to the above claim: that more than one phage able to infect the same target bacterium is required to combat treatment resistance via enhancement of cocktail depth of activity. This exception occurs when cocktail phages are supplied which target phage-resistant mutants but not the parental, primary bacterial target [166,221,222,223]. This is a phenomenon that might be described as the equivalent of phage resistance resulting in the ‘sensitization’ of a bacterium to a different phage type, as analogous to phage resistance resensitizing a bacterium to a given antibiotic type (Section 4.2). Smug et al. [162] have described such a phage combination instead as a “Cocktail of sequentially infecting phages”. Note that this is not equivalent to serially applying phages (Section 3), since cocktails by definition consist of phages that are applied in parallel. Rather, these are simultaneously dosed phages that are not able to simultaneously target the initially infecting bacterial strain.

By contrast are anti-treatment-resistance phage cocktails that simultaneously target both parental and resistant mutant bacteria, as emphasized in Section 4.4.1. Smug et al. [162] describe this approach as using a “Cocktail of parallelly infecting phages”. In this case, phages are both applied and act in parallel. Thereby these cocktails simultaneously infect across a bacterial population rather than being applied in parallel but acting only in serial (i.e., “Sequentially infecting”). For examples of cocktails consisting of “Parallelly infecting phages” as designed explicitly to combat treatment resistance, see Li et al. [78] or Chen et al. [219]. They generated cocktails using additional phages that intentionally targeted resistant bacterial mutants, but which also targeted the parental bacterium.

Sequentially infecting approaches [162], along with those of Li et al. [78] or Chen et al. [219], can be viewed as employing what essentially is an autophage-like process of phage acquisition (Section 3.1). The only difference is that actual autophages are generated against clinically isolated bacterial resistant mutants. By contrast, in this section, emphasis is especially placed on the pre-clinical isolation of such bacterial mutants, hence ‘Proactive autophages’.

The sequentially infecting cocktail considered by Smug et al. [162] specifically involves phage resistance that is mediated via alterations in bacterial capsule production. One has to wonder, though, to what extent such strategies may be broadly effective. That is, whether sets of sequentially infecting phages making up a cocktail may be sequentially effective in treating a diversity of potential bacterial strains, beyond those strains used to develop specific approaches. Indeed, this is a potential drawback that Smug et al. point out.

#### 4.4.3. Breadth of Depth (Empirical Anti-Treatment-Resistance Phage Cocktails)

**Figure 4:**
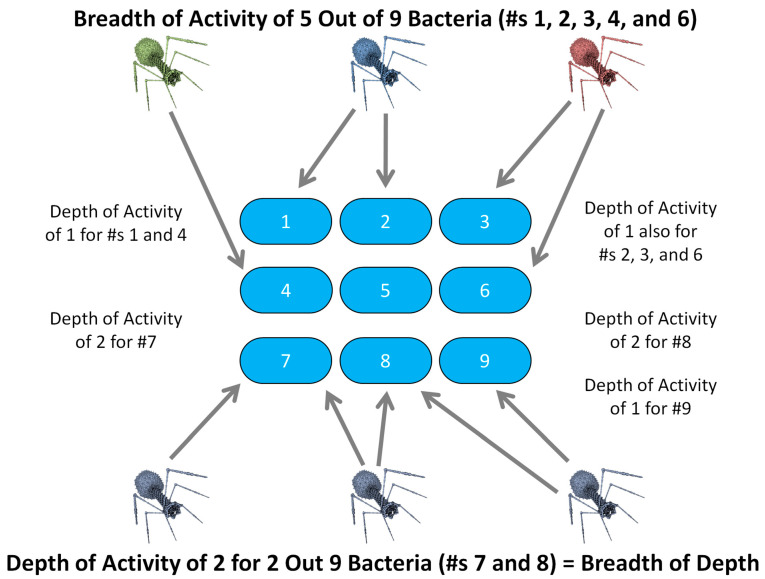
Breadth, depth, and breadth of depth of antibacterial activities. The middle nine shapes represent different bacterial strains while the six virions symbolize six different phages, each possessing a different functional diversity. The top row of virions demonstrate breadth of activity, in this case with the free phages targeting 5 of the 9 bacterial strains present (~56%), but each bacterial strain is targeted by only a single phage (the breadth of depth for a depth of 1 therefore is also 5). The bottom row of virions demonstrate a breadth of activity of 3 (#s 7, 8, and 9 for 33%) but also a depth of activity of 2 for two of the bacteria (#s 7 and 8); the latter indicates that each of those two bacteria are being targeted by two different types of phages. The resulting breadth of depth for a depth of 2 is therefore also 2 bacteria out of 9 (22%), while the breadth of depth for a depth of 1 is 3 (#s 7, 8, and 9). If three bacteria were each within the host range of at least two different phage types from a single phage cocktail, then the breadth of depth—for a depth of 2—would instead be 3. However, this scenario was not illustrated here to avoid excessive figure complexity.

It is also possible to create traditional phage cocktails that possess depths of activity against a multitude of bacterial strains, even without explicit cocktail development against phage-resistant bacterial mutants. These would be cocktails containing different combinations of phages, each drawn from separate cross-resistance groups, e.g., [90]. These are cocktails which collectively are able to target with different phages more than one different bacterial strain (depth > 1). This is a so-called ‘breadth of depth’ (Section 2.3; Figure 4). Such breadth of depth can be achieved without all cocktail phages being able to target the same bacterial strains, just so long as multiple bacterial types are impacted by multiple phage types.

We have designed a relatively simple algorithm that is useful for characterizing such cocktails in terms of their breadth of depth, as based on phage host range data [49]. This is available for use online, either taking [224] or not taking [215] cross-resistance into account. For example, a cocktail could target perhaps half of bacterial strains to a depth of 2 or more, implying a breadth, at that depth, of 50%, or what in Abedon et al. [49] dubbed “Breadth_2_”. The meaning of the latter is that “half of the bacteria tested were individually hit by at least two different phages sourced from two different cross-resistance groups, while the other half were not.” For other algorithms that may be used for generating phage cocktails, see [47,209,216,217] and see also [162].

### 4.5. Phage–Antibiotic Combination Therapies

By mixing multiple therapeutics into simultaneous treatments, mutations to resistance to one treatment can be immediately countered by another, already applied treatment. That, of course, represents the underlying basis for employing phage cocktails to combat treatment resistance—cocktails possessing depths of activity that are greater than one for their target bacteria. Such combination therapies, but not based on phages, are commonly used in anti-cancer treatments, anti-human immunodeficiency virus (antiretroviral) therapies, or antibiotic treatments against certain bacteria, particularly *Mycobacterium tuberculosis* [225,226,227,228,229]. A crucial advantage of providing mixtures of treatments simultaneously, rather than serially, is that the separate components can prevent further in situ target evolution (cancer cell, virus, or bacterium). This is evolution that otherwise can occur during a grow-back of mutants that have become resistant to a single applied agent. See Table 8 for a summary of pros and cons associated with this combination therapy approach.

#### 4.5.1. Combating Not Just Treatment Resistance

Increasingly, phage treatments are being undertaken in combination with antibiotics. This is recommended for clinical practice [17] and has been the case for many clinical phage therapy case studies [15]. It is being carried out, at least in part, as a means of combating treatment resistance to either agent [5,155,176,178,182,230,231,232,233,234,235]. Phage–antibiotic combination treatments, however, also should have a high potential for broadening spectra of activity toward addressing community resistance. This means that already antibiotic-resistant bacteria encountered during empirical treatments can, at the same time, be at least possibly sensitive to treatment phages. Similarly, antibiotics can serve as a backup for circumstances where targeted bacteria are not impacted by applied phages.

This also is a situation where the antibiotic antagonism of phage infection activity [236,237] should be less relevant. Such antagonism generally results from interference with the metabolic activities of phage-hosting bacteria, but this should be less of a factor given bacterial antibiotic resistance. Thus, toward combating community resistance, phages and antibiotics should complement each other’s antibacterial activities. And they should be able to do so without substantially interfering with those activities, as phages generally should not antagonize antibiotic actions while antibiotics should antagonize phages only if bacteria are antibiotic-susceptible.

#### 4.5.2. Mostly Avoids Cross-Resistance

An important advantage of phage–antibiotic combination therapies is that bacterial targets of phages and antibiotics tend to substantially differ. These are, for instance, bacterial surface molecules for phages vs. specific aspects of cell wall synthesis, ribosomes, etc., for antibiotics. Thus, generally, bacteria will need two independent mutations to become resistant to combinations of treatment phages and treatment antibiotics.

This suggestion, though, comes with the caveat that in certain circumstances bacterial cross-resistance to phages and antibiotics has indeed been reported [75,153,238,239,240,241,242]. As a further caveat, and as noted, many antibiotics can antagonize phage infection activities, such as a phage’s ability to produce new virions or to overcome bacterial anti-phage defense mechanisms [236,237,243]. Again, though, should bacteria mutate to resistance to a treatment antibiotic, then this antagonism should substantially decline. This is suggestive, however, of a need to supply phages in multiple doses over time, to assure the continuing presence of sufficient phage numbers as an ongoing guard against bacterial mutation to antibiotic resistance.

#### 4.5.3. Combating Also Antibiotic Resistance

The primary consideration here has been on strategies that reduce the impact of bacterial evolution of phage resistance during therapy, i.e., of treatment resistance. Phage actions, however, also can interfere with bacterial evolution of antibiotic resistance. This could occur due to (i) phages reducing bacterial populations in numbers (thereby there being fewer bacteria present to mutate to antibiotic resistance, e.g., see Section 4.1 for an equivalent argument), (ii) phages targeting antibiotic-resistant bacteria, and/or (iii) phage resistance resulting in increased sensitivity of bacteria to antibiotics (Section 4.2). Note also (iv) the ability of phages to target antibiotic-tolerant persister cells [59,148,236,244] and (v) the potential for phage–antibiotic synergy [232,234,245,246,247,248]. Combining phages with antibiotics thus has the potential to maintain the ongoing utilities of both phages and antibiotics.

#### 4.5.4. Antibiotics as Back up Treatment

An interesting alternative to phage–antibiotic combination therapy is presented by Fujiki et al. [178]. They suggest starting with phages which, in selecting for phage resistance, also increase antibiotic sensitivity (Section 4.2.3). But then, rather than dosing with phages and antibiotics in parallel, they suggest following up phage-only application with an appropriate antibiotic solely if phage treatment alone has been unsuccessful in clearing the bacterial infection. This approach has the advantage of potentially successfully treating a bacterial infection while avoiding antibiotic application, but without abandoning altogether the anti-resistance potential of antibiotic use. The caveat, though, is that this represents a serial application of antibacterial agents (Section 3). Phage-resistant bacterial populations, should they grow back, could therefore potentially give rise to bacteria that are both phage- and antibiotic-resistant.

## 5. Conclusions

It is of course possible to combine antimicrobial treatments. With phages, this is often carried out to expand overall spectrum of activity breadth, so as to overcome what is described here as community resistance. In most clinical cases, however, antimicrobial or anti-cancer combination treatments are utilized instead to limit the potential for targeted microorganisms or cancer cells to evolve resistance. That is, to inhibit their displaying treatment resistance. This latter contrasting strategy we have described elsewhere, in a phage therapy context, as treatments providing a greater spectrum of activity depth [49]. The underlying basis of achieving this greater depth is to impact more than one vulnerable aspect of a target organism.

This depth can be achieved by combining phages with other phages (as cocktails; Section 4.4) or instead by combining phages with antibiotics (Section 4.5). In either case, this works so long as cross-resistance to the different agents is rare. Avoiding cross-resistance, as well as antagonism between agents, may be achieved with certain phage types that individually are able to target two different aspects of a bacterium at once. In particular, this can be accomplished by showing an affinity for more than one receptor molecule found on a bacterium’s surface (Section 4.3). Alternatively, phages can be chosen that reduce bacterial virulence should those bacteria evolve resistance (Section 4.2). As an important caveat to these various efforts note, again from Baym et al. [50] (p. 6), that “even if a treatment strategy can suppress the evolution of resistance, it is unlikely to be widely adopted clinically unless it also provides increased survival on a per-patient basis.”

If a given phage type or even a phage cocktail is either unable or no longer able to impact a given bacterial infection, then it will be necessary to apply a different antibacterial agent, such as a new phage. These new phages can be obtained via their isolation against the otherwise phage-resistant bacterial etiology (as autophages; Section 3.1), by choosing a new phage from an ideally already well-characterized phage collection (a phage bank; Section 3.2), by modifying an existing phage genetically (phage training or engineering; Section 3.3), or perhaps even by applying a new but previously chosen second phage, or antibiotic (Section 4.5.4). All of these phage-substitution strategies in principle can be effective; this comes, though, at the expense of treatment delays. Serial application of treatments also can reduce anti-evolution efficiency relative to simultaneous impacts, this reduction in efficiency being due to regrowth of bacterial populations prior to phage substitution.

These various approaches—particularly autophages, phage banks, and phage training and/or engineering—nonetheless in principle can also be used as a basis of phage cocktail generation to minimize bacterial evolution of resistance. This is rather than using approaches solely to respond to that evolution as it occurs in the course of phage treatment, i.e., in response to treatment resistance.

## Figures and Tables

**Table 1:** Pros and cons of employing autophages to combat treatment resistance.

Description	Pros	Cons
New-phage isolation using bacterial strains infecting a patient as isolation hosts	Phage host range specifically includes the targeted infection etiology	Requires time and expertise; In addressing treatment resistance, it is reactive rather than proactive; Can permit substantial replication of phage-resistant bacteria prior to phage substitution

**Table 2:** Pros and cons of the use of phage banks to combat treatment resistance.

Description	Pros	Cons
Previously isolated phage stocks are available for phage substitution	Phage characterization can take place ahead of time, allowing relatively rapid and safe phage substitution	Requires access to a phage bank, though phage crowdsourcing could serve as an alternative phage source; In addressing treatment resistance, it is reactive rather than proactive; Can permit substantial replication of phage-resistant bacteria prior to phage substitution

**Table 3:** Pros and cons of using phage training to combat treatment resistance.

Description	Pros	Cons
In vitro evolution of a treatment phage especially toward new host range properties	Phages modified through mutation may require only minimal further characterization; Phages can also be modified in highly targeted manners using molecular techniques (phage engineering)	Requires time and expertise; In addressing treatment resistance, it is reactive rather than proactive; Can permit substantial replication of phage-resistant bacteria prior to phage substitution; Trained phages can possess similar or identical immunological characteristics to parental phages

**Table 4:** Pros and cons of minimizing bacterial growth to combat treatment resistance.

Description	Pros	Cons
Rapidly bringing bacterial infections under control prior to their growing in cell number to a point where resistance mutations are present	Ideally, prevents mutations to resistance from occurring; Is by necessity proactive relative to the occurrence of resistance mutations	Unless treatments are prophylactic, or bacterial infections otherwise are caught very early, then this approach can be difficult or impossible to successfully implement

**Table 5:** Pros and cons of targeting bacterial fitness determinants to combat treatment resistance.

Description	Pros	Cons
Intentional selection by treatment phages for bacterial mutants that are unable to continue to support ongoing disease	Allows initiation of anti-treatment-resistance strategies with monophages; Combats bacterial evolution of phage resistance by harnessing natural selection; Can be proactive rather than reactive	Evidence is needed on a per-phage basis that reductions in bacterial fitness are seen across multiple potentially targeted bacterial strains; May not be as effective given bacterial infections of immunocompromised individuals

**Table 6:** Pros and cons of using phages that recognize multiple receptors to combat treatment resistance.

Description	Pros	Cons
Certain phages are able to adsorb using different receptor molecules displayed by the same bacterial strains	Allows initiation of anti-treatment-resistance strategies with monophages; Two independent mutations may be required of bacteria to achieve phage resistance rather than just one mutation; Can be proactive rather than reactive	It is uncertain how many phages possess this property; It is uncertain what fraction of bacterial hosts found within a phage’s host range will normally display both phage receptors

**Table 7:** Pros and cons of employing phage cocktails to combat treatment resistance.

Description	Pros	Cons
Combination therapy involving only phages (in principle, though, phage cocktails can also be combined with non-phage antibacterial agents such as antibiotics)	Can prevent substantial growth of bacteria that have mutated to phage resistance; Can be proactive rather than reactive	Requires multiple phage types, each able to impact a targeted bacterium; Requires a low potential for bacteria to mutate to cross-resistance to those multiple phage types; Potential for phage antagonism; Greater cost and complexity

**Table 8:** Pros and cons of combining phages with antibiotic to combat treatment resistance.

Description	Pros	Cons
Therapy involving phage combination especially with an antibiotic	Can prevent substantial growth of phage-resistant bacteria; Two independent mutations in most cases are required of bacteria to achieve co-resistance rather than just one mutation; Can be proactive rather than reactive	Antibiotics can be antagonistic to phage infection abilities; Antibiotics can possess side effects that would tend to be absent given treatments solely with phages; Any observed efficacy will be difficult to assign to phage action alone

## Appendix A. Meanings of Autophage

Documented in this appendix is the use of “autophage” as found especially in the recent English-language literature as well as providing some historical context. Distinguished especially are (i) phage isolations against a specific bacterium but not necessarily isolating the phage from that same environment in which the targeted bacterium was isolated vs. (ii) phage isolations from the same environment in which a targeted bacterium had been isolated. See especially [69], p. 55, where it is noted that, “The term autobacteriophage appears to have originally referred to the isolation of a phage from the patient [thus, ‘From’]. Currently, the term has also been used to refer to phages isolated from the environment [thus, ‘Against’]…”

From that same reference, note specifically a suggestion (perhaps) that isolating phages from the same environment in which a targeted bacterium was isolated (i.e., “From”) is not necessarily an ideal strategy. Particularly, as translated there from d’Hérelle [69] (from 1938, p. 56, with emphasis theirs):

…why try to prepare an auto-bacteriophage which will certainly be inferior, because the strain of bacteriophage will be not selected among hundreds of others, but simply found at random and therefore of variable virulence? And if, *a contrario*, the ‘coli- phage’ remains without activity against the bacterium infecting a given patient, barring a most remote chance of one in a thousand, it is pointless to try the preparation of an auto-bacteriophage because it would take months of assiduous research to discover a bacteriophage able to attack the resistant bacterial isolate.

See Table A1 for further documentation of how “Autophage” has been used in the relatively recent phage therapy literature.

**Table A1:** Descriptions of “autophage” in the recent English-language phage therapy literature.

Year	Ref.	Quotation	Isolated *
2023	[249]	“bacteriophages isolated from the same environment as the pathogen” (p. 9 and citing [250])	From
2022	[251]	“personalized phage (autophages) or standard formulation (fixed phage cocktails)” (p. 552)	Against?
2022	[252]	“phages can be obtained or isolated from the patient where the pathogenic agent is found, calling this virus autochthonous phage or autophage” (p. 105 and citing, in part, [250])	From
2021	[253]	“A custom phage (autophage) was prepared… that was fully sensitive against the *S. mitis* isolated from the patient’s sample.” (p. 5)	Against
2021	[37]	“In cases when patients’ strains are not susceptible to the commercially available preparations, or if their infection is caused by an entirely different organism (on a species or genus level), an individualized phage preparation—custom phage ^†^—is offered. Such tailored bacteriophages are targeted at specific strains that have been isolated and identified in patients’ biological samples.” (p. 3); “This is when she ordered her first autophage.” (p. 7)	Against?
2020	[84]	“the auto-phage preparation which is personalised for an individual patient” (p. 307)	Against
2018	[254]	“i.e., auto-phage specifically manufactured for the use of a particular patient” (p. 5)	Against
2018	[35]	“a tailored strategy by training phages on the patient’s strain as soon as it became available in a form of highly personalized medicine. Of note, this latter strategy is applied at the Eliava Institute in the process of development of so-called ‘autophages’.” (p. 7)	Against ^‡^
2018	[250]	“autophage (bacteriophage isolated from the same environment where the pathogen is isolated)” (p. 4367 and citing [255] elsewhere which we can speculate is for the statement there, “When no active phage is present against a severe pathogen, the lytic phage may be found, isolated directly from environment”, though that reference [255] does not appear to actually use the term, “autophage”, nor “same” in front of “environment”)	From
2011	[256]	“we isolate specific ‘autophage’ against patient’s specific bacteria” (p. 646)	Against
2011	[63]	“Sometimes custom phage preparations are developed for a patient’s infection (autophage), a procedure that usually takes a few days to weeks.” (p. 936)	Against?
2010	[257]	“under extreme circumstances new ‘auto-phages’ may be isolated from environmental sources, using the patient’s own bacteria to select them” (p. 71 and see [69] as the citation)	Against
2009	[258]	“In problem cases, new phage specific to the patient’s bacteria are occasionally isolated from sewage, amplified and sent to the hospital; these are called ‘autophage’.” (p. 265)	Against

* ”Against” a specific etiology vs. “From” the same environment, referring to a phage isolated using a specific bacterial etiology as host vs. a phage isolated from a specific patient. See the main text of this appendix for additional discussion of the difference. Question marks indicate uncertainty as to the authors’ intentions. ^†^ It is unclear why or how the term “autophage” is being used in this study as the word is present only once vs. 72 times for “custom phage”. Replacing autophage with custom phage is being avoided here, however, due to the use of custom phage with potentially alternative meanings (Section 3.1.2). ^‡^ It is difficult with this study to distinguish between an autophage isolated “Against” vs. an autophage as a trained phage.
